# Prevalence and Surgical Implications of Cystic Artery Variations in Calot’s Triangle: A Prospective Observational Study

**DOI:** 10.7759/cureus.108224

**Published:** 2026-05-04

**Authors:** Bhagavat G Kothule, Ajit M Dikle, Kirankumar R Vairage

**Affiliations:** 1 Department of General Surgery, Government Medical College and Hospital, Dharashiv, IND

**Keywords:** calot’s triangle, critical view of safety, cystic artery variations, hepatobiliary anatomy, laparoscopic cholecystectomy

## Abstract

Background

Variations in the cystic artery within Calot’s triangle are clinically significant during laparoscopic cholecystectomy (LC), as unrecognized vascular anomalies may increase the risk of hemorrhage and bile duct injury. This study aimed to evaluate the prevalence and surgical implications of cystic artery variations in terms of origin, number, position, and location.

Methods

This prospective observational study was conducted at Amar Hospital, Patiala, from 2023 to 2025 and included 100 patients undergoing LC. Intraoperative findings were recorded regarding cystic artery origin, number (single/double/absent), positional relationship to the cystic duct, location relative to Calot’s triangle, and the presence of Moynihan’s hump. Operative duration and blood loss were also documented. Data were analyzed using GraphPad Prism, and the Chi-square test was applied for categorical variables.

Results

The mean age was 53.5 ± 13.81 years, with a female predominance (77%). The cystic artery originated from the right hepatic artery in 93% of cases, with variations arising from the gastroduodenal artery (5%), common hepatic artery (1%), and left hepatic artery (1%) (p < 0.001). A single cystic artery was observed in 96% of patients, while 4% had double arteries (p < 0.001). The artery was most commonly located superomedial to the cystic duct (93%) and within Calot’s triangle (92%) (p < 0.001). Moynihan’s hump was noted in 2% of cases. The mean operative time was 53.5 ± 13.81 minutes, and the mean blood loss was 19.9 ± 5.05 mL.

Conclusion

Although classical cystic artery anatomy predominates, significant variations exist. Careful identification of the cystic artery and adherence to the Critical View of Safety are essential to minimize intraoperative complications.

## Introduction

Laparoscopic cholecystectomy (LC) is the gold standard treatment for symptomatic cholelithiasis and inflammatory gallbladder diseases worldwide. Although it is considered a safe and routinely performed procedure, vascular and biliary injuries remain significant complications, particularly during dissection of Calot’s triangle [1-3]. The incidence of bile duct injury during LC has been reported to be higher than that in open cholecystectomy during the early learning curve, emphasizing the importance of precise anatomical identification before ligation of structures [4, 5]. Among the anatomical components of Calot’s triangle, the cystic artery demonstrates considerable variability in its origin, number, course, and relationship to adjacent biliary structures, thereby increasing the risk of hemorrhage and inadvertent ductal injury [6, 7].

Classically, the cystic artery arises from the right hepatic artery and courses within Calot’s triangle; however, multiple studies have demonstrated substantial deviations from this pattern. Variations include origin from the left hepatic artery, common hepatic artery, gastroduodenal artery, or aberrant right hepatic artery, as well as double or absent cystic arteries [8-10]. Ding YM et al. classified cystic artery variations into Calot’s triangle type, outside-Calot’s-triangle type, and compound type, highlighting their surgical relevance [6, 11]. Similarly, Andall RG et al., in a comprehensive review, reported that nearly 20% of cases demonstrate atypical positioning or multiple cystic arteries, which may lead to operative difficulty and an increased risk of vascular injury [7, 12, 13]. The presence of Moynihan’s hump (caterpillar hump) of the right hepatic artery further complicates the operative field and has been associated with a short cystic artery and a higher bleeding risk [14, 15].

Given the unpredictable nature of vascular anatomy in the hepatobiliary region, thorough intraoperative identification and achievement of the Critical View of Safety (CVS) are essential to minimize complications [16-18]. Awareness of the prevalence and patterns of cystic artery variations is particularly important for surgeons in training and for reducing conversion rates and operative morbidity. Therefore, the present prospective study aims to evaluate the prevalence of vascular anatomical variations of the cystic artery in terms of origin, number, position, and location within Calot’s triangle during LC. Hence, this study was undertaken to assess the prevalence and surgical implications of cystic artery variations during LC.

## Materials and methods

This prospective observational study was conducted in the Department of General Surgery at a tertiary care hospital in Patiala from 2023 to 2025. Patients were included using consecutive sampling. The sample size for the present study was calculated based on the expected proportion of the cystic artery originating from the right hepatic artery, which has been reported to be approximately 80-90% in previous studies [6]. Assuming an expected prevalence (p) of 85%, with a 95% confidence level (Z = 1.96) and an absolute precision (d) of 7%, the sample size was calculated using the standard formula for estimation of a single proportion: n = Z² × p(1-p) / d². Substituting the values yielded a sample size of approximately 100 patients. A total of 100 patients undergoing LC for symptomatic cholelithiasis, acute or chronic cholecystitis, gallbladder polyp, or acalculous cholecystitis were included. Patients aged 15-85 years were eligible for inclusion. Pregnant patients, cases converted to open cholecystectomy, and those with contraindications to laparoscopy or general anesthesia were excluded. The study protocol was approved by the Institutional Ethics Committee (IEC) of Amar Hospital, Patiala (IEC/SRC Ref No: DNB/2023/02; IEC meeting dated 08.02.2023), and written informed consent was obtained from all participants prior to enrollment.

All patients underwent standard preoperative evaluation, including complete blood count, liver and renal function tests, coagulation profile, viral markers, abdominal USG, and other investigations as clinically indicated. LC was performed under general anesthesia using a standard four-port technique. After establishing pneumoperitoneum, Calot’s triangle was meticulously dissected to obtain the CVS. The cystic artery was identified intraoperatively, and its variations were documented with respect to origin, number (single, double, or absent), position relative to the cystic duct (superomedial, posterolateral, anterior, or inferior), location (inside or outside Calot’s triangle), and the presence of Moynihan’s hump. All findings were recorded in a predesigned proforma.

The primary variables included the origin, number, position, and location of the cystic artery. Secondary variables included operative duration and intraoperative blood loss. Efforts were made to minimize observer bias by recording intraoperative findings using a standardized proforma. Data were entered into Microsoft Excel and analyzed using GraphPad Prism (version 10.1.2). Categorical variables were expressed as frequencies and percentages, while quantitative variables were presented as mean ± SD or median with interquartile range, as appropriate. The Chi-square test was applied for comparison of categorical variables, and a p-value of ≤0.05 was considered statistically significant. This study has been reported in accordance with the STROBE guidelines.

## Results

Table 1 shows that the study population (n=100) had a mean age of 53.5 ± 13.81 years, with a median age of 54 years (IQR: 42.8-64) and a range of 23-85 years, indicating a predominantly middle-aged to elderly cohort. Females constituted the majority of patients, 77 (77%), while males accounted for 23 (23%). The most common indication for surgery was chronic calculus cholecystitis (85%), with acute and complicated gallbladder conditions contributing to a smaller proportion of cases. Regarding comorbidities, half of the patients (50%) had no associated medical conditions, whereas hypertension (27%) and diabetes mellitus (16%) were the most prevalent comorbidities, followed by hypothyroidism (6%) and other less frequent conditions. In terms of surgical history, most patients (74%) had no prior surgeries. Among those with previous procedures, gynecological surgeries were the most common (26%), followed by abdominal (5%), hepatobiliary (4%), urological (3%), neurosurgical (1%), and other surgeries (2%). Overall, the findings indicate that LC was predominantly performed in female patients with chronic gallbladder disease, with a relatively low comorbidity burden and limited prior surgical history.

**Table 1:** Baseline characteristics of study population (n = 100). n: Number; %: Percentage; CCC: Chronic calculus cholecystitis; ACC: Acute calculus cholecystitis; ACCC: Acute-on-chronic calculus cholecystitis; GB: Gallbladder; CVA: Cerebrovascular accident; IHD: Ischemic heart disease; C-section: Cesarean section; ERCP: Endoscopic retrograde cholangiopancreatography; CBD: Common bile duct; TURP: Transurethral resection of the prostate.

Variable	Value
Age (years)	
Mean ± SD	53.5 ± 13.81
Median (IQR)	54 (42.8-64)
Range	23-85
Gender	
Male, n (%)	23 (23.0%)
Female, n (%)	77 (77.0%)
Indication for surgery, n (%)	
Chronic calculus cholecystitis (CCC)	85 (85.0%)
Acute calculus cholecystitis (ACC)	3 (3.0%)
Acute-on-chronic calculus cholecystitis (ACCC)	3 (3.0%)
Empyema of the gallbladder	3 (3.0%)
Cholelithiasis	2 (2.0%)
Chronic calculus cholecystitis with gallbladder polyp	1 (1.0%)
Gangrenous gallbladder	1 (1.0%)
Gallbladder polyp	1 (1.0%)
Mucocele of the gallbladder	1 (1.0%)
Medical history	
No comorbidity	50 (50.0%)
Hypertension	27 (27.0%)
Diabetes mellitus	16 (16.0%)
Endocrine (hypothyroidism)	6 (6.0%)
Psychiatric disorders	10 (10.0%)
Respiratory disorders	3 (3.0%)
Cardiovascular/CVA/IHD	4 (4.0%)
Others	4 (4.0%)
Surgical history	
No previous surgery	74 (74.0%)
Gynecological (C-section, hysterectomy, tubectomy)	27 (27.0%)
Hepatobiliary (ERCP/CBD stenting)	4 (4.0%)
Abdominal (appendectomy, laparotomy, hernia repair)	5 (5.0%)
Urological (nephrostomy, TURP, varicocele)	3 (3.0%)
Neurosurgical	1 (1.0%)
Others	2 (2.0%)

Table 1 shows that 50 (50%) patients had no associated medical comorbidities. Hypertension was the most common comorbidity, observed in 27 (27%) patients, followed by diabetes mellitus in 16 (16%). Other comorbidities included hypothyroidism in 6 (6%) patients, cardiovascular conditions such as cerebrovascular accident (CVA) and ischemic heart disease (IHD) in 4 (4%), respiratory disorders in 3 (3%), psychiatric disorders in 10 (10%), and other conditions in 4 (4%) patients. With respect to surgical history, the majority of patients, 74 (74%), had no prior surgical procedures. Among those with previous surgeries, gynecological procedures were the most common, reported in 27 (27.0%) patients, followed by abdominal surgeries in 5 (5%), hepatobiliary interventions such as endoscopic retrograde cholangiopancreatography (ERCP) with common bile duct (CBD) stenting in 4 (4%), urological procedures in 3 (3%), neurosurgical procedures in 1 (1%), and other surgeries in 2 (2%) patients.

The cystic artery most commonly originated from the right hepatic artery in 93 (93%) cases. Variations included origin from the gastroduodenal artery in 5 (5%) cases, the common hepatic artery in 1 (1%), and the left hepatic artery in 1 (1%). No cases of origin from an aberrant right hepatic artery or other sources were observed. The distribution of cystic artery origin was statistically significant (χ² = 2.47, p < 0.001). With respect to the number of cystic arteries, a single cystic artery was identified in 96 (96%) patients, whereas double cystic arteries were observed in 4 (4%) cases; no patient had an absent cystic artery. This distribution was also statistically significant (χ² = 96.00, p < 0.001) (Table 2).

**Table 2:** Anatomical variations of the cystic artery. RHA: Right hepatic artery; GDA: Gastroduodenal artery; CHA: Common hepatic artery; LHA: Left hepatic artery; CT: Calot’s triangle.

Variable	Origin / Category	Frequency	Chi-square	p-value
Origin of cystic artery	Right hepatic artery (RHA)	93 (93.0%)	2.47	<0.001
	Gastroduodenal artery (GDA)	5 (5.0%)		
	Common hepatic artery (CHA)	1 (1.0%)		
	Left hepatic artery (LHA)	1 (1.0%)		
	Aberrant right hepatic artery	0 (0.0%)		
	Other	0 (0.0%)		
Number of cystic arteries	Single	96 (96.0%)	96	<0.001
	Double	4 (4.0%)		
	Absent	0 (0.0%)		
Position of cystic artery relative to cystic duct	Superomedial	93 (93.0%)	160.22	<0.001
	Posterolateral	4 (4.0%)		
	Anterior	3 (3.0%)		
	Inferior	0 (0.0%)		
Location of cystic artery	Inside Calot’s triangle	92 (92.0%)	155.42	<0.001
	Outside Calot’s triangle	8 (8.0%)		
Moynihan’s hump	Present	2 (2.0%)	142.5	<0.001
	Absent	98 (98.0%)		

Regarding the positional relationship of the cystic artery to the cystic duct, the superomedial position was the most frequent, observed in 93 (93%) patients, followed by the posterolateral position in 4 (4%) and the anterior position in 3 (3%) patients; no cases of inferior positioning were noted. This distribution was statistically significant (χ² = 160.22, p < 0.001). In terms of location within Calot’s triangle, the cystic artery was located inside the triangle in 92 (92%) patients, while in 8 (8%) patients, it was found outside the triangle, which was statistically significant (χ² = 155.42, p < 0.001). Moynihan’s hump was absent in the majority of cases, being absent in 98 (98%) patients and present in only 2 (2%) patients, demonstrating a statistically significant distribution (χ² = 142.5, p < 0.001).

Table 3 shows that the mean duration of surgery was 53.5 ± 13.81 minutes, with a median operative time of 54 minutes (IQR: 42.8-64 minutes), and the duration ranged from 23 to 85 minutes. The mean intraoperative blood loss was 19.9 ± 5.05 mL, with a median of 20 mL (IQR: 17-23 mL), and the observed blood loss ranged from 10 to 35 mL. Overall, the operative duration and blood loss values indicate that LC was performed with minimal intraoperative morbidity in the study population.

**Table 3:** Duration of surgery and blood loss.

Parameter	Mean ± SD	Median (IQR)	Range
Duration of surgery (minutes)	53.5 ± 13.81	54 (42.8-64)	23-85
Blood loss (mL)	19.9 ± 5.05	20 (17-23)	10-35

## Discussion

The present study evaluated vascular anatomical variations of the cystic artery during LC and demonstrated that the cystic artery most commonly originated from the right hepatic artery in 93 (93%) cases. This finding is consistent with previous studies, in which the right hepatic artery was reported as the predominant source of origin in the majority of cases. Ding YM et al. reported the classical right hepatic artery origin in 85.5% of cases [6], while Andall RG et al., in a systematic review of over 9,800 cases, found right hepatic origin in approximately 79-81% of patients [7]. Similarly, Pandey P et al. (2024) and Gupta R et al. (2023) reported the right hepatic artery as the most common origin in more than 90% of cases [4, 5]. In our study, variations arising from the gastroduodenal artery in 5 (5%) cases, the common hepatic artery in 1 (1%) case, and the left hepatic artery in 1 (1%) case were also observed, comparable to the findings described by Suzuki M et al. [9] and Chen TH et al. [19]. Such atypical origins are clinically significant, as failure to identify them may increase the risk of vascular injury and intraoperative hemorrhage.

With respect to the number of cystic arteries, a single cystic artery was identified in 96 (96%) patients, while double cystic arteries were observed in 4 (4%) cases. These findings align with those reported by Schiewe JA et al., who observed a single cystic artery in 91 (91%) cases [14], and Singh H et al., who reported single arteries in approximately 92% of patients undergoing LC [8]. The presence of multiple cystic arteries, although less frequent, remains surgically important because unrecognized accessory branches may lead to troublesome bleeding. In terms of positional relationship, the superomedial course of the cystic artery relative to the cystic duct was most common in 93 (93%) cases, which is consistent with observations by Muhammad Samir Irfan Deflaoui T et al., who described superomedial positioning as the predominant anatomical pattern [20].

The majority of cystic arteries in the present study were located within Calot’s triangle in 92 (92%) patients, similar to the distribution reported by Harpreet Singh H et al. [8] and Abeysuriya V et al. [21], who documented an inside-triangle prevalence ranging from 80% to 89%. These findings emphasize the importance of meticulous dissection within Calot’s triangle and adherence to the CVS, as described by Strasberg SM and Brunt LM [16], to minimize bile duct and vascular injury. The operative duration and intraoperative blood loss in our study were within acceptable limits, suggesting that awareness of vascular variations and careful surgical technique contribute significantly to safe LC. Overall, although classical anatomy was most common, anatomical variations were not infrequent, underscoring the need for vigilance during hepatobiliary surgery.

## Conclusions

The present study demonstrates that the cystic artery most commonly originates from the right hepatic artery and is usually present as a single vessel located within Calot’s triangle in a superomedial position. Although classical anatomy predominates, clinically significant variations in origin, number, and course do occur and may increase the risk of vascular injury during LC if unrecognized. Careful dissection, thorough knowledge of hepatobiliary anatomy, and adherence to the CVS are essential to ensure safe surgical outcomes and minimize intraoperative complications.
